# The LPS-induced neutrophil recruitment into rat air pouches is mediated by TNFα: likely macrophage origin

**DOI:** 10.1080/09629359791479

**Published:** 1997-12

**Authors:** C-D. Arreto, C. Dumarey, M-A. Nahori, B. B. Vargaftig

**Affiliations:** 1 Unité de Pharmacologie Cellulaire Institut Pasteur 25, rue du Dr Roux Paris Cedex 15 75724 USA; 2 Département de Pharmacologie Faculté de Chirurgie Dentaire Université René Descartes Paris V France

## Abstract

The role of resident cells during the lipopolysaccharide (LPS)-induced neutrophil recruitment into rat air pouches was investigated. In this model, LPS (*Escherichia coli*, O55: B5 strain; 2–2000 ng) induced a dose– and time-dependent neutrophil recruitment accompanied by the generation of a tumour necrosis factor-α (TNFα)-like activity. Dexamethasone (0.05–5 mug) and cycloheximide (6 ng), injected 2 h before LPS into the pouches, inhibited the neutrophil recruitment and the generation of the TNFα-like activity, while the H1-receptor antagonist mepyramine (1 and 4 mg/kg, i.p., 0.5 h before LPS) and the PAF-receptor antagonist WEB 2170 (0.05 and 1 mg/kg, i.p., 0.5 h before LPS) had no effect. Purified alveolar macrophages (AM) were used to replenish the pouches of cycloheximide-treated recipient rats. AM provided by PBS-treated animals led to the recovery of the LPS-induced neutrophil recruitment and of the TNFα-like formation contrasting with those from cycloheximide-treated animals (1 mg/kg, i.p.). When delivered *in situ*, liposome-encapsulated clodronate, a macrophage depletor, significantly impaired both the LPSinduced neutrophil recruitment and the TNFα-like activity. An anti-murine TNFα polyclonal antibody (0.5 h before LPS) was also effective. These results emphasize the pivotal role of macrophages for LPS-induced neutrophil recruitment via the formation of TNFα.


Mediators of Inflammation, **6**, 335–343 (1997)

### The LPS-induced neutrophil recruitment into rat air pouches is mediated by TNFα: likely macrophage origin

C-D. Arreto[1,2,CA], C. Dumarey[1], M-A. Nahori[1] and B. B. Vargaftig[1]

[1]Unité de Pharmacologie Cellulaire, Institut Pasteur; 25, rue du Dr Roux, 75724 Paris Cedex 15; [2]Département de Pharmacologie, Faculté de Chirurgie Dentaire, Université René Descartes, Paris V, France

[CA]Corresponding Author
Tel: (+33) 45 68 86 82
Fax: (+33) 45 68 87 03
Email: ⟨vargaftig@pasteurfr⟩

**The role of resident cells during the lipopolysaccharide (LPS)-induced neutrophil recruitment into rat air pouches was investigated. In this model, LPS (*Escherichia coli*, O55: B5 strain; 2–2000 ng) induced a dose- and time-dependent neutrophil recruitment accompanied by the generation of a tumour necrosis factor-α (TNFα)-like activity. Dexamethasone (0.05–5 μg) and cycloheximide (6 ng), injected 2 h before LPS into the pouches, inhibited the neutrophil recruitment and the generation of the TNFα-like activity, while the H1-receptor antagonist mepyramine (1 and 4 mg/kg, i.p., 0.5 h before LPS) and the PAF-receptor antagonist WEB 2170 (0.05 and 1 mg/kg, i.p., 0.5 h before LPS) had no effect. Purified alveolar macrophages (AM) were used to replenish the pouches of cycloheximide-treated recipient rats. AM provided by PBS-treated animals led to the recovery of the LPS-induced neutrophil recruitment and of the TNFα-like formation contrasting with those from cycloheximide-treated animals (1 mg/kg, i.p.). When delivered *in situ*, liposome-encapsulated clodronate, a macrophage depletor, significantly impaired both the LPS-induced neutrophil recruitment and the TNFα-like activity. An anti-murine TNFα polyclonal antibody (0.5 h before LPS) was also effective. These results emphasize the pivotal role of macrophages for LPS-induced neutrophil recruitment via the formation of TNFα.**

**Key words:** Rat air pouch, Lipopolysaccharide, Neutrophil recruitment, Macrophage, Tumour necrosis factor-α

### Introduction

Leukocyte recruitment[1,2] and the formation of pro-inflammatory mediators, including different cytokines, are the hallmark of an inflammatory response. The latter is characterized by vasodilatation, rapidly followed by neutrophil adhesion to endothelium and migration into the perivascular connective tissue.

*In vitro* studies have shown that the bacterial endotoxin (lipopolysaccharide, LPS) is not by itself a chemotactic factor,[3] even though LPS may interact with neutrophils via CD14 and the LPS-binding protein (LBP) to express CR3 activity which mediates neutrophil adhesion.[4,5] In fact, local cell targets seem to be more relevant *in vivo* and in particular, resident macrophages are believed to play a pivotal role in the recognition and the transduction of the effects of LPS (for review, see Manthey and Vogel[6]) since they produce the chemotactic mediators IL-1α and β, TNFα, IL-8 and MIP-1 and 2 as well as $LTB_4$ and PAF. Furthermore, LPS elicits a selective and transient cycloheximide-dependent collagenase synthesis by macrophages, providing thus another potential tissue damaging factor.[7]

Initially described as a potent inflammatory cytokine derived from LPS-activated macrophages, TNFα may also account for neutropenia, neutrophilia[8,9] and neutrophil recruitment[2,10–12] and activation.[13,14] TNFα also induces the expression of cell surface molecules, leading to adherence on endothelial cells.[15] As an early cytokine, TNFα is released extracellularly within 15 min after its gene transcription upon exposure to inflammatory stimuli.[16] Its broad spectrum of activities and the amounts produced by macrophages stimulated by various products (up to 2% of their total biosynthesis) suggest that TNFα is an important mediator of LPS-induced inflammatory response.[17] Glucocorticoids such as dexamethasone inhibit the production of circulating TNFα in mice, rats[18] and guinea-pigs[19] treated with LPS. Dexamethasone also alters the phagocytic functions of

© 1997 Rapid Science Publishers macrophages *in vitro*[20] and contributes to protect rat macrophages against LPS-induced TNFα production *in vitro*, even though less so *in vivo*.[21]

The cutaneous air pouch provides a virtual cavity which can be tailored as a migration chamber covered innerly with a lining membrane, the facsimile synovium.[22] Since the walls of the pouch are formed by macrophages and fibroblasts and a few mastocytes, we took advantage of this model to study the mode of action of LPS. Because of the potential damage engendered by the neutrophil towards connective tissue,[23] we investigated the dependency of LPS-induced neutrophil emigration upon resident cells and bring evidence that macrophages and TNFα are respectively the target and the mediator of neutrophil recruitment following LPS injection into rat air pouches.

### Materials and Methods

### Animals

Brown-Norway rats (200–250 g) (Iffa Credo, France) were allowed to take food and drink *ad libitum* at room temperature.

### Rat air pouch

The air pouch was induced according to Edwards *et al.*[22] At day 0, rats were anaesthetized with ketamine (50 mg/kg, i.m.) and their dorsum was thoroughly shaved and gently disinfected with ethanol 70°. Syringes and needles were one-purpose material. Twenty ml of sterile air taken under a laminar flux hood were injected subcutaneously with a gauge 26G × 0.5"; 0·45 × 12 needle; thus, disruption of the underlying cutaneous connective tissue allowed to make an air-full cavity. At day 5, 10 ml of sterile air were injected in similar conditions in order to maintain pouch patency. At day 7, the pouches were injected with LPS (2–2000 ng/ml) under a volume of 1 ml. Control animals were injected with the same volume of the LPS vehicle, i.e. phosphate buffered saline (PBS) without $Ca^{2+}$ nor $Mg^{2+}$.

### Neutrophil migration assessment

At various times (2 h, 24 h, 48 h and 96 h) after LPS injection, animals were killed with an overdose of sodium penthiobarbitone. Four ml of heparinized PBS solution (5 IU/ml) were injected into the pouch in order to wash the cavity by a gentle massage. The washing solution which was recovered at over 95% was collected into 6 ml-polypropylene round bottom tubes (Falcon° 2063, sterile/gamma irradiated) stored on an ice bath.

Injection of the mastocyte degranulating agent compound 48/80, under 1 ml (250 μg/ml) into the rat air pouch followed the same procedures. This concentration was chosen for its ability to induce granulocyte infiltration in the mouse skin.[24]

Leukocyte and differential counts were performed with a cell counter (Coulter©) and a Cytospin (Hettich Universal©), respectively, and allowed to calculate the total number of leukocyte neutrophils recovered.

One ml aliquots prepared from the pouch washing were centrifuged (400 g, 10 min at 4°C), pellets and aliquoted supernatants being kept at −40°C until further analysis.

### TNFα assay

TNFα production was determined directly in the supernatant using the TNFα sensitive cell line WEHI-164. Cells were plated out in 96-well plates ($8 \times 10^4$ cells/50 μl/well) and the samples (50 μl/well) or the human recombinant TNFα (hr-TNFα) standards ($1-10^6$ U/ml hr-TNFα, 50 μl/well) were added. After 24 h incubation (37°C, 7.5% $CO_2$), MTT (tetrazolium salt, Sigma, USA) was added (0·125 mg/well). After an incubation of 4 h, the cells were lysed with buffer (20% sodium dodecyl sulphate (SDS) in 50% N,N-dimethylformamide (DMF), pH 4.7, 100 μl/well) for 18 h. The difference of absorbances measured at 550 nm and 630 nm with an ELISA reader (Dynatech MR5000) allowed to evaluate the TNFα-like activity.

### Modulation of neutrophil recruitment

In some experiments, cycloheximide (6 ng/ml) or dexamethasone (0·5–5 μg/ml) were injected into the pouch 2 h before LPS (200 ng/ml) under a volume of 1 ml. In other experiments, animals received mepyramine (1–4 mg/kg) i.p. 30–45 min before LPS was injected into the pouch.

An anti-murine TNFα immunoglobulin preparation was prepared as follows. Female HY/CR rabbits (2500 g; Charles River, St Aubin les Elboeufs, France) were immunized at 2 weeks intervals by three injections of reduced murine rTNFα (Immungenex, Los Angeles, CA, USA) emulsified in adjuvant (Hunter Titermax; CytRx Co., Norcross, Germany): the first one with 50 μg, the second and the third with 25 μg. The animals were bled 2 weeks after the last injection, and total immunoglobulins were obtained after precipitation with 40% saturation of ammonium sulphate. Purified murine polyclonal anti-TNFα (2·5 mg/ml) was administered into the rat air pouches in a volume of 0.7 ml, 0.5 h before LPS. Control animals were treated under same conditions with the same amounts of preimmune immunoglobulins.

### Macrophage replenishment

Rats were anaesthetized (sodium penthiobarbitone, 60 mg/kg, i.p.), the trachea was cannulated and broncho-alveolar lavages (BAL) with sterile saline under a volume of 6 ml were performed. This procedure was repeated until a final volume of 36 ml was obtained in a 50 ml-polypropylene graduated conical tube (Falcon© 2098, Blue Max, sterile/gamma irradiated) on ice bath. Immediately after centrifugation (400 g, 5 min at 4°C), the cell suspension underwent an hypotonic lysis with sterile water to remove remaining erythrocytes. Then, cells were counted (Counter Coulter©) and the suspension was diluted to a final concentration of $10^6$ and $10^4$ cells per ml. Purity and viability were assessed by differential count and blue trypan dye exclusion, respectively.

In other experiments, rats were treated with cycloheximide i.p. (1 mg/kg, 0.5 ml).[25] Control animals received similar volumes of saline. After 2 h, the animals were sacrificed with an overdose of penthiobarbitone and both groups underwent the same procedure to purify the alveolar macrophages.

### Liposome preparation and experimental design

Multilamellar liposomes were prepared according to Van Rooijen and Van Nieuwmegen.[26] In brief, 75 mg dipalmitoylphosphatidylcholine and 11 mg cholesterol were dissolved in chloroform in a round bottom flask. The thin film that formed on the walls after rotary evaporation at 45°C was dispersed by gentle shaking for 10 min in 10 ml of PBS (pH 7.4), in order to prepare empty liposomes, and in 10 ml of a solution of 2 g clodronate (dichloromethylene diphosphonate or $Cl_2MDP$) in PBS, in order to prepare liposomes with encapsulated $Cl_2MDP$. The preparation was kept for 2 h at room temperature and sonicated four times for 5 min at 45°C in a waterbath (50 Hz) and kept at room temperature for a further 2 h. Then, liposomes were filtered through 1.2-μm Minisart NML filters (disposable syringe holders, sterile, pyrogenfree, hydrophilic, Sartorius, Germany) centrifuged at 100 000 g to 0.5 h, finally resuspended in 5 ml PBS and kept at 4°C.

Rats with 7-day-old air pouches were anaesthetized and injected into this preformed cavity with either 0.3 ml liposome-encapsulated clodronate or 0.3 ml liposome-encapsulated PBS (empty liposome) for 96 h before LPS stimulation as previously described.

In order to overcome the cell counter inability to differentiate the remaining injected liposomes and the LPS-recruited cells, 100 μl aliquots of pouch washing were allowed to stretch on glass slides by cytocentrifugation. After a Diff-Quik® staining, the slides were observed under light microscope at magnification ×1000. A differential leukocyte count was performed (neutrophil, eosinophil, mononuclear cell) taking account of the total fields observed. Thus, the data was expressed as the number of neutrophils per field.

### Materials

Lipopolysaccharide from *Escherichia coli* strain O55: B5 was purchased from Difco (Detroit, MI, USA); heparin from Choay (Paris, France); ketamine hydrochloride (KETALAR©) stored at 4°C as a 100 mg/ml stock solution was from Parke-Davis (Courbevoie, France); sodium penthiobarbitone was from Sanofi Santé Animale (Libourne, France); compound 48/80, bovine serum albumin, tetrazolium salt (MTT), the histamine-receptor antagonist H1 pyrilamine maleate (mepyramine), dexamethasone phosphate, chloroform, dipalmitoylphosphatidylcholine and cholesterol were from Sigma (St Louis, MO, USA); PAF-receptor antagonist WEB 2170 was a kind gift of Boehringer Ingelheim (Germany), cycloheximide was from Merck (Darmstadt, Germany); Diff-Quik® kit was purchased from Baxter S.A. (Maurepas, France), clodronate disodium salt (dichloromethylene diphosphonate, $Cl_2MDP$) was a kind gift of Boehringer Mannheim GmbH (Germany); murine recombinant TNFα was purchased from Immungenex (Los Angeles, CA, USA); human recombinant TNFα was a kind gift of Dr G. R. Adolf (Wien, Austria); WEHI-164 cells were a kind gift of Dr I. L. Bonta (Rotterdam, The Netherlands).

### Data analysis

Experimental value are given as mean ± SEM. Statistical significance of differences between two means of data were evaluated by a Students's *t*-test for unpaired observations and *P*-values less than 0.05 were considered to be significant.

### Results

### Dose-effect relationship induced by LPS compared with compound 48/80

LPS (2–2000 ng, 2 h) induced a dose-dependent leukocyte recruitment (Fig. 1a) which was predominantly formed by neutrophils (LPS: $67 \pm 5{\cdot}7\%$ vs. control: $14{\cdot}6 \pm 7{\cdot}6\%$, $P < 0{\cdot}001$, $n = 5$–$7$) (Fig. 1b). The threshold dose of LPS for inducing a significant leukocyte infiltration was 20 ng ($P < 0{\cdot}05$, $n = 7$) which correlated with the enhancement of the neutrophil population at this dose ($P < 0{\cdot}01$, $n = 7$). Since leukocyte and neutrophil infiltration plateaued at 200 ng, this dose was chosen for further experiments. By contrast, compound 48/80 (250 μg, 2 h) failed to elicit leukocyte infiltration, compared with the vehicle.

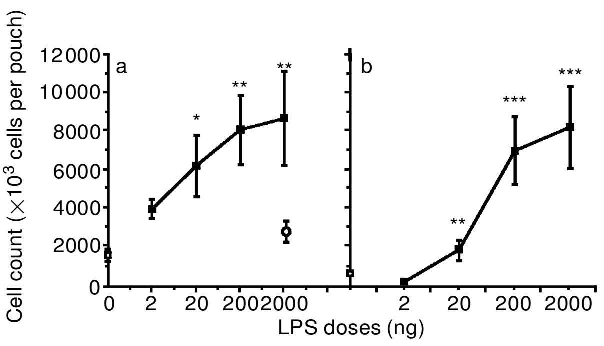

FIG. 1. Dose-dependent leukocyte and neutrophil recruitment induced by LPS (■) but not by compound 48/80 (○) into rat air pouches. LPS, at the doses of 2, 20, 200, 2000 ng was injected under a volume of 1 ml and samples removed at 2 h. Compound 48/80 at the dose of 250 μg and PBS was tested under the same volume and at the same time-point. Responses are for (a) leukocyte and (b) neutrophil recruitment and are expressed as mean ± SEM of total number of cells per pouch for leukocytes ($n = 5$–$7$) and neutrophils ($n = 5$–$7$) in LPS-treated animals (■) and in PBS-treated animals (□) (* $P < 0.05$, ** $P < 0.01$, *** $P < 0.001$).

### Time-course of leukocyte and neutrophil recruitment induced by LPS

Leukocyte recruitment by LPS (200 ng) peaked at 2 h and 24 h (Table 1) ($P < 0{\cdot}01$, $n = 7$) and was over at 48–96 h. Since at 2 h cell infiltration was predominantly constituted by neutrophils (Table 1) ($P < 0{\cdot}01$, $n = 7$), this time point was chosen for subsequent studies.

### Detection of TNFα-like activity in supernatants of pouch

Injected at the dose of 200 ng (2 h), LPS induced the generation of a significant TNFα-like activity detected in the pouch washings ($P < 0{\cdot}01$, $n = 5$–$9$) under conditions where compound 48/80 (250 μg/ml, 2 h) failed to do so (Fig. 2).

### Interference of dexamethasone with LPS-induced neutrophil recruitment and TNFα-like activity, failure of mepyramine and WEB 2170

Injected 2 h before LPS, dexamethasone (50–5000 ng) inhibited dose-dependently the LPS-induced recruitment of neutrophils ($P < 0{\cdot}01$ for the dose of 5000 ng, $n = 7$) (Fig. 3). Accordingly, the threshold for inhibition was between 1000 and 2500 ng. By contrast, the H1-receptor antagonist mepyramine (1 and 4 mg/kg, i.p., 0.5 h before LPS) and the PAF-receptor antagonist WEB 2170 (0.05 and 1 mg/kg, i.p., 0.5 h before LPS) failed to interfere with the LPS-induced recruitment of neutrophils (Tables 2 and 3).

Injected 2 h before LPS, dexamethasone (5000 ng) also inhibited the generation of TNF-α like activity ($P < 0{\cdot}05$, $n = 6$) (Fig. 3, inset).

**Table 1**. Time-course of the LPS-induced leukocyte and neutrophil recruitment into rat air pouches. Leukocyte and neutrophil recruitment was evaluated at different time-points (2 h, 24 h, 48 h and 96 h) for the dose of 200 ng of LPS injected into the air pouches under a volume of 1 ml. Results are expressed as mean ± SEM for $n = 5$–$7$ experiments (** $P < 0.01$)

| | Time (h) | | | |
|---|---|---|---|---|
| | 2 | 24 | 48 | 96 |
| Leukocytes (×10³ cells) | | | | |
| LPS | 8500 ± 1970** | 7500 ± 700** | 1000 ± 120 | 2730 ± 60 |
| Control | 1990 ± 370 | 3200 ± 1300 | 1470 ± 140 | 2500 ± 680 |
| Neutrophils (×10³ cells) | | | | |
| LPS | 7290 ± 1840** | 1850 ± 350** | 20 ± 10 | 260 ± 70 |
| Control | 300 ± 180 | 310 ± 160 | 0 | 250 ± 150 |

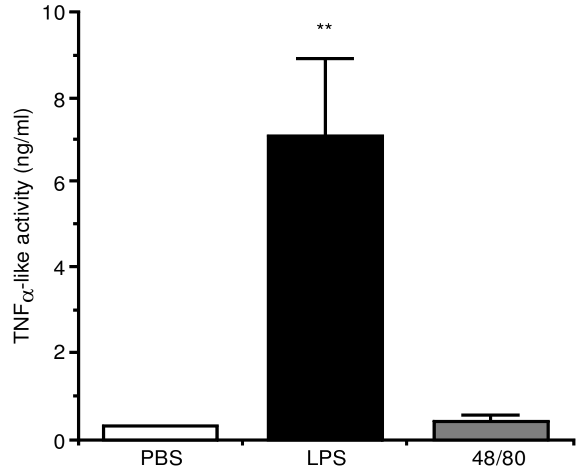

FIG. 2. Ability of LPS (200 ng, 2 h) and of compound 48/80 (250 μg, 2 h) to generate the TNFα-like activity in the rat air pouch. The TNFα-like activity was evaluated for LPS and compound 48/80 from the supernatants of pouch washing. Results are expressed as mean ± SEM of TNFα-like activity concentration for $n = 4$ in the PBS group (□), $n = 9$ in the LPS group (■) and $n = 5$ in the compound 48/80 group (▩). (* $P < 0.05$, ** $P < 0.01$).

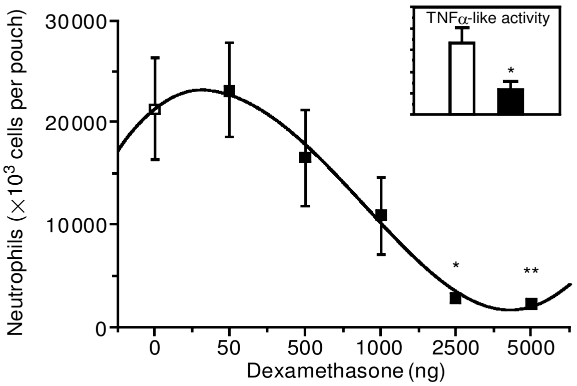

FIG. 3. Effects of dexamethasone (DEX) injected into the rat air pouches on neutrophil recruitment and TNF-α like activity induced by LPS. DEX at the doses of 50, 500, 1000, 2500 and 5000 ng (■) and the vehicle (0 ng) (□) were tested under a volume of 1 ml, 2 h before LPS (200 ng, 2 h). Neutrophil responses are expressed as mean ± SEM of 5–7 animals per dose. TNF-α like activity (inset) was assessed for the dose of 5 μg and expressed as mean ± SEM of TNF-α like activity concentrations recovered in the air pouch washing supernatants for 5 animals (* $P < 0.05$, ** $P < 0.01$).

Table 2. Failure of mepyramine on LPS-induced recruitment of leukocytes and neutrophils into rat air pouches. The effects of mepyramine (1 and 4 mg/kg, i.p., 0.5 h before LPS) on neutrophil recruitment were evaluated for the dose of 200 ng of LPS (2 h, into the pouches). Results are expressed as mean ± SEM of total number of cells per pouch for $n = 5$ experiments. No result was statistically different from the control group

| | Mepyramine (4 mg/kg) | Mepyramine (1 mg/kg) | Control (Saline) |
|---|---|---|---|
| Leukocytes ($\times 10^3$ cells) | 5500 ± 1400 | 8850 ± 2690 | 5440 ± 900 |
| Neutrophils ($\times 10^3$ cells) | 5070 ± 1390 | 8820 ± 2400 | 5080 ± 880 |

Table 3. Failure of WEB 2170 to inhibit LPS-induced recruitment of leukocytes and neutrophils into rat air pouches. The effects of WEB 2170 (0.05 and 1 mg/kg, i.p., 0.5 h before LPS) were evaluated for the dose of 200 ng of LPS (2 h, into the pouches). Results (mean ± SEM of total number of cells per pouch, $n = 5$) of each single experiment are reported. No result was statistically different from the control LPS group

| | WEB 2170 (50 mg/kg) | WEB 2170 (1 mg/kg) | Control (Saline) |
|---|---|---|---|
| Leukocytes ($\times 10^3$ cells) | 10600 ± 1230 | 11900 ± 2600 | 10300 ± 2800 |
| Neutrophils ($\times 10^3$ cells) | 7700 ± 1760 | 8900 ± 2800 | 8900 ± 2400 |

### Interference of cycloheximide with LPS-induced neutrophil recruitment and generation of TNFα-like activity

Injected into the pouch at 6 ng, 2 h before 200 ng of LPS, cycloheximide inhibited significantly the LPS-induced neutrophil recruitment (Fig. 4) ($P < 0{\cdot}01$, $n = 5$), but it was inactive, when injected together with LPS ($P > 0{\cdot}05$, $n = 5$). Similarly, cycloheximide interfered with the TNFα-like activity generated by LPS (Fig. 4, inset) ($P < 0{\cdot}05$, $n = 5$).

### Replenishment of cycloheximide-treated pouches with alveolar macrophage (AM) restores the LPS-induced recruitment of neutrophils and the accompanying generation of TNFα-like activity

Alveolar macrophages (AM) (purity: 90%; viability: 95%) were adjusted to a final concentration

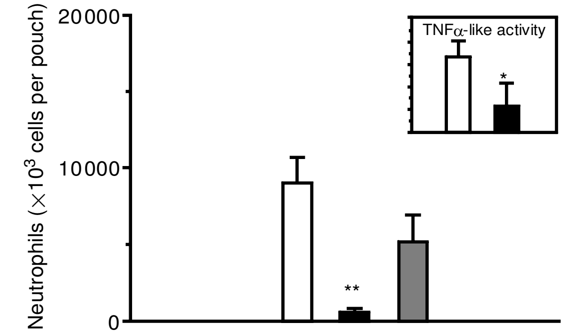

FIG. 4. Interference of cycloheximide (6 ng) injected into the rat air pouch 2 h before LPS with both neutrophil recruitment and TNF-α like activity. Cycloheximide was injected under a volume of 1 ml with PBS, either 2 h before or together with LPS (200 ng). Responses are for neutrophils and TNF-α like activity (box). Results are expressed as mean ± SEM of total number of cells per pouch ($n = 5$) for the recruitment and of TNF-α like activity concentrations recovered in the air pouch washing supernatants for $n = 5$. Cycloheximide-treated animals, 2 h befere LPS (■), together with LPS (▩) and vehicle-treated animals (□) (* $P < 0.05$, ** $P < 0.01$).

of $10^6$ and $10^4$ cells per ml and injected into the air pouch previously treated with cycloheximide (6 ng per pouch, 2 h before). This replenishment restored significantly the LPS-induced neutrophil recruitment when compared with cycloheximide-treated pouches or with PBS stimulation (Fig. 5). In addition, replenishment with $10^4$ AM per ml from cycloheximide-treated animals failed to support the LPS-induced neutrophil recruitment (Fig. 6), contrasting to AM from control animals ($P < 0.001$ for neutrophils, $n = 6$).

Replenishment with $10^6$ AM/ml also restored the generation by LPS of TNFα-like activity in the pouch. Indeed, the replenished animals generated 13·02 ± 3 ng/ml of TNFα-like activity upon LPS injection, whereas cycloheximide-treated animals generated 2·4 ± 2·4 ng/ml ($n = 5$; $P < 0.05$) (Fig. 5, inset). By contrast, replenishment with $10^4$ AM/ml generated 4·06 ± 0·4 ng/ml of TNFα-like activity, a value not significant different from that obtained with AM from cycloheximide-treated animals (Fig. 6, inset).

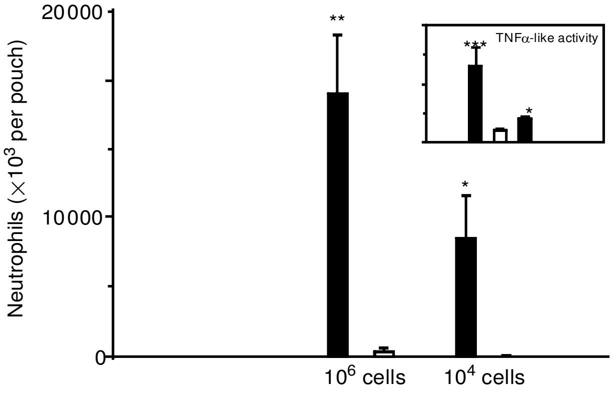

FIG. 5. Effects of replenishment with alveolar macrophages on LPS-induced recruitment of neutrophils and TNF-α like activity in rat air pouches. For the recruitment, air pouches were pretreated with 6 ng of cycloheximide for 2 h, then replenished with $10^6$ macrophages for four animals or with $10^4$ macrophages for six animals under a volume of 1 ml for 0.5 h, then stimulated with LPS (200 ng) or PBS for 2 h under a volume of 1 ml. Results are expressed as mean ± SEM of total number of cells. For the TNF-α like activity (inset), the same conditions were used and results are expressed as mean ± SEM of this activity recovered in the air pouch washing supernatants. Comparison for each replenishment group was performed for LPS (■) vs. PBS (□) (* $P < 0.05$, ** $P < 0.01$, *** $P < 0.001$).

### Interference of liposome-encapsulated clodronate with LPS-induced neutrophil recruitment and generation of TNFα-like activity in rat air pouches

Liposome-encapsulated clodronate, injected into the rat air pouches (300 μl, 96 h before LPS), produced a marked inhibition of LPS-induced neutrophil emigration (Fig. 7) and generation of TNFα-like activity (Fig. 7, inset) in rat air pouches, when compared with empty liposomes (control: 57·1 ± 11·9 neutrophils per field; 20·6 ± 13 ng/ml TNFα-like activity; treated: 10·3 ± 1·7 neutrophils per field; 2·4 ± 0·5 ng/ml TNFα-like activity; $n = 5$–6, $P < 0.01$ and $P < 0.01$ respectively).

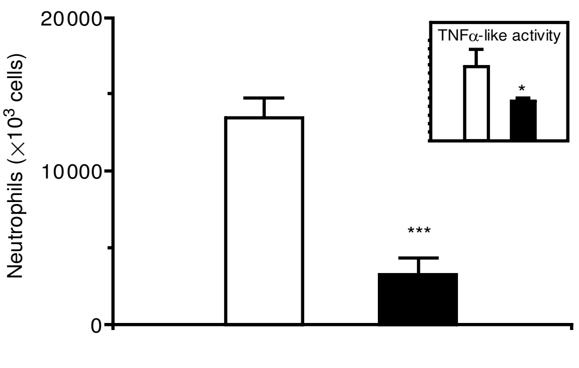

FIG. 6. Effects of cycloheximide (CHX) (1 mg/kg, i.p. 2 h before LPS) with the restoration of LPS-induced neutrophil recruitment and of following replenishment with alveolar macrophages in rat air pouches. Animals were treated with CHX or its vehicle, i.p. for 2 h before bronchoalveolar lavage. Replenishment was performed with $10^4$ macrophages (1 ml) for 0.5 h, then LPS or PBS stimulation were tested (200 ng, 2 h, 1 ml). Results are expressed as mean ± SEM of total number of cells for $n = 6$ experiments for the recruitment and of the TNFα-like activity (inset) recovered in the air pouch washing supernatants for $n = 6$ under the same conditions. Comparison was performed for CHX (■) vs. its vehicle (□) (** $P < 0.01$, *** $P < 0.001$).

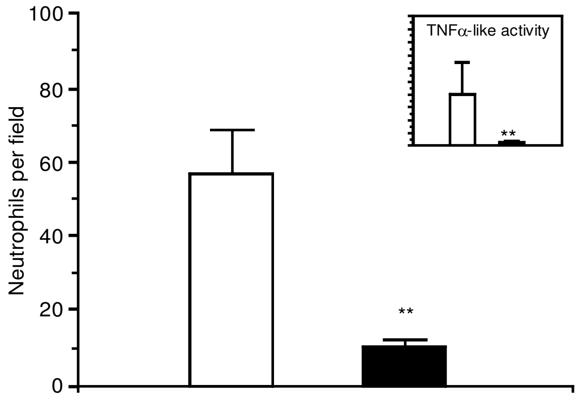

FIG. 7. Effects of liposome-encapsulated clodronate on neutrophil recruitment and the TNF-α like activity triggered by LPS in Brown Norway rat air pouches. Liposome-encapsulated clodronate and empty liposomes were administered under a volume of 0.3 ml into the 7-day-old air pouches, 96 h before LPS (200 ng, 2 h). Results are expressed as mean ± SEM of number of neutrophils per field ($n = 6$) and of the TNF-α like activity recovered in the air pouch washing supernatants ($n = 6$) (inset). Comparison was performed for liposome-encapsulated clodronate (■) vs. empty liposome (□) (** $P < 0.01$; *** $P < 0.001$).

### Anti-mouse TNFα polyclonal antibodies suppressed the LPS-induced neutrophil recruitment and the generation of TNFα-like activity in rat air pouches

Under conditions where both LPS-induced neutrophil recruitment and TNFα-like activity were significantly inhibited by anti-mouse TNFα polyclonal antibody (0.7 ml into the pouch, 0.5 h before LPS), a treatment with non-immune polyclonal antibodies was uneffective (control: $15611 \pm 4236$ $10^3$ neutrophils vs. treated: $3888 \pm 411$ $10^3$ neutrophils, $P < 0{\cdot}05$ (Fig. 8); control: $7{\cdot}3 \pm 1{\cdot}2$ ng/ml of TNFα-like activity vs. treated: $0{\cdot}11 \pm 0{\cdot}08$ ng/ml of TNFα-like activity, $P < 0{\cdot}01$ (Fig. 8, inset; for $n = 4$ animals).

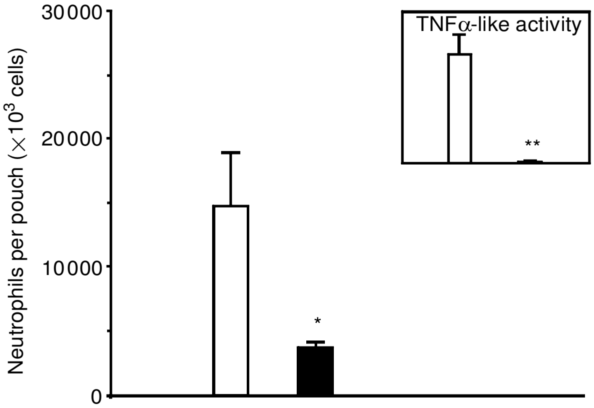

FIG. 8. Effects of the mouse polyclonal anti-TNFα antibody with neutrophil recruitment and TNFα-like induced by LPS. Results are expressed as mean ± SEM of total number of neutrophils per pouch and of TNFα-like activity concentrations recovered in the air pouch washing supernatants. The treated group received into their pouch 0.7 ml of the polyclonal antibody for 0.5 h, then LPS (□). The control group was treated with a non-immune antibody preparation under same conditions (■). Results are expressed as mean ± SEM of total number of cells for four experiments for the recruitment and of the TNF-α like activity (inset) recovered in the air pouch washing supernatants for four experiments. Comparison was performed for treated group (■) vs. control group (□) (* $P < 0.05$, ** $P < 0.01$).

### Discussion

The injection of LPS into the rat air pouch resulted in a potent time- and dose-dependent recruitment of neutrophils. Suppression of this recruitment by low amounts of the protein synthesis inhibitor cycloheximide and by dexamethasone, injected into the pouch, suggested the involvement of a local target, capable of producing a secondary mediator. Since the concomitant injection of cycloheximide and LPS failed to block neutrophil recruitment, the 2 h interval required for inhibition is probably accounted for by the time needed for inhibition of protein synthesis. Alternatively, cycloheximide may induce apoptosis,[27,28] an active process requiring protein and RNA synthesis[29] and involving the degradation of nuclear DNA.[30] Although apoptosis is prevented in most cells

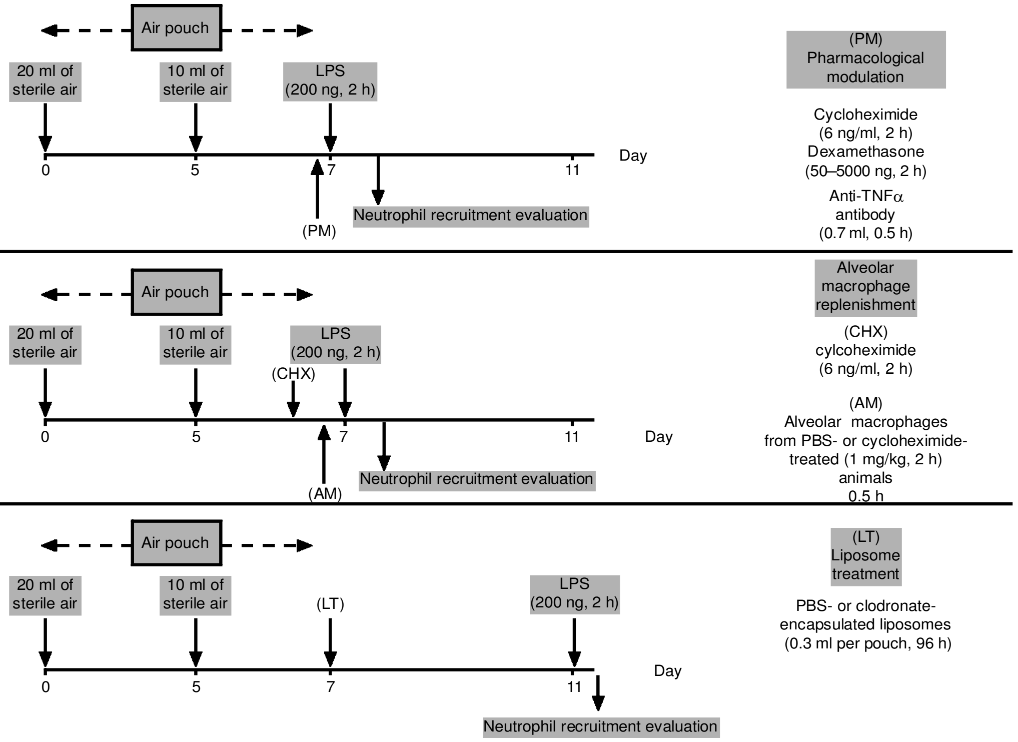

FIG. 9. Flow sheet protocol.

by cycloheximide, HL-60 cells,[31] hepatocytes,[25,27] thymocytes and macrophages[28] undergo apoptosis when incubated with micromolar doses of this drug. However, apoptosis is unlikely to account for the suppression of neutrophil recruitment in our experiments, since the conditions used here (2 h, 6 ng $\approx$ 200 nmol) were previously shown not to induce apoptosis.[28]

Resident mast cells might be affected by cycloheximide and by dexamethasone during LPS-induced neutrophil emigration, as suggested by the results of Matsuda *et al.*[24] with murine air blebs, a model closely resembling an air pouch but lacking a facsimile synovium. Similarly, Tannenbaum *et al.*,[32] injected compound 48/80 into the rat skin at doses 50 times below ours and provoked a marked neutrophilia within 1–2 h. Compound 48/80 also caused mast cell degranulation, neutrophil adhesion and emigration into the cheek pouch vasculature.[33] Nevertheless, mast cell involvement is unlikely in our model, since the compound 48/80 failed to induce a significant cell infiltration or generation of TNFα-like activity, throughout all time intervals. A more likely hypothesis is that fibroblasts and macrophages of the pouch lining tissue account for the LPS-induced production of chemotactic substances and the consequent neutrophil recruitment, in agreement with the anatomopathological studies of Edwards *et al.*[22] To verify to what extent this might apply to our model, alveolar macrophages were used to replenish the air pouch and indeed inhibition by cycloheximide of neutrophil emigration and generation of TNFα-like activity, was surmounted by transferring fresh alveolar macrophages from control animals, whereas macrophages from cycloheximide-treated animals were not effective, as reported.[12,34] The use of alveolar macrophages rather than other sources of macrophages is supported by practical considerations concerning macrophage purification and their ability to provide large amounts of TNFα[35] or chemoattractants such as MIP-1α.[36]

Liposomes are particularly efficient in delivering water-soluble drugs into phagocytic cells, since phagocytosis is followed by phospholipase-induced disruption of the liposome phospholipid bilayers and the release of entrapped drugs. In particular, liposome-encapsulated clodronate which should deplete air pouch macrophages[37,38] reduced significantly LPS-induced neutrophil recruitment. Since in separate experiments murine peritoneal macrophages were still absent after 5 days treatment (data not shown), the participation of the air pouch macrophage during neutrophil emigration is thus likely. Clodronate is specific for macrophages, since it neither affected the neutrophil population nor other cell types.[37,39,40]

Since a potential role for histamine and PAF in LPS-induced neutrophil recruitment into rat air pouches was excluded by selective antagonists, the effectiveness of cycloheximide and dexamethasone strongly suggests the involvement of protein synthesis-dependent mediators such as TNFα. As mentioned, the hypothesized participation of mast cells as a major source of preformed TNFα[41] was ruled out by the failure of compound 48/80 to induce neutrophil recruitment and detectable TNFα-like activity. Among cells that express the TNF gene, the macrophage is unique, insofar as it is capable of secreting-1000 times more TNFα in response to LPS than any other cell type.[42,43] Furthermore, a TNFα-like activity was detected in the air pouch transplanted with alveolar macrophages from naive, but not from cycloheximide-treated animals. In addition, the rat alveolar macrophage was shown to be an important producer of chemoattractants when stimulated by LPS.[44] Taken together, these results suggest that TNFα-like activity accounts for the LPS-induced neutrophil recruitment in our model. This was supported by the efficacy of an anti-murine polyclonal TNFα antibody to abrogate both LPS-triggered neutrophil recruitment and TNFα-like activity. The resident macrophage is the likely source of this cytokine, as its formation was prevented by both *in situ* treatments with liposome-encapsulated clodronate and polyclonal antibody. Nevertheless, our results do not clarify whether TNFα acts as a direct chemoattractant to promote neutrophil emigration or if an intermediary chemokine is required, such as CINC/GRO or MIP-1 or 2. Indeed, even though TNFα itself may increase adhesion molecules such as CD11/18 to promote neutrophil emigration,[45] several studies seem to plead for a chemokine networking involving either epithelial cell[46] or endothelial cell.[47]

In summary, we suggest that neutrophil recruitment induced by LPS is a macrophage-dependent event, involving the *de novo* synthetized TNFα which act directly or via secondary mediators.

### References

1. Issekutz AC, Bhimji S. Role of endotoxin in the leukocyte infiltration accompanying *Escherichia coli* inflammation. *Infect Immun* 1982; **36**: 558.
2. Cybulsky MI, McComb DJ, Movat HZ. Neutrophil leukocyte emigration induced by endotoxin. Mediator roles of IL-1 and TNFα. *J Immunol* 1988; **140**: 3144–3149.

3. Hugli T. Chemotaxis. *Curr Opin Immunol* 1989; **2**: 19–27.
4. Wright SD, Ramos RA, Hermanowski A, Rockwell P, Detmers PA. Activation of the adhesive capacity of CR3 on neutrophils by endotoxin: dependence on lipopolysaccharide binding protein and CD14. *J Exp Med* 1991; **173**: 1281–1286.
5. Weingarten R, Sklar LA, Mathison JC, Omidi S, Ainsworth T, Simon S, Ulevitch RJ, Tobias PS. (1993) Interactions of lipopolysaccharide with neutrophils in blood via CD14. *J Leukoc Biol* 1993; **53**: 518–524.
6. Manthey CL, Vogel SN. Interaction of lipopolysaccharide with macrophages. In: Zwilling BS, Eisenstein TK (eds) *Macrophage-Pathogen Interactions*. New York–Basel–Hong Kong: Marcel Dekker, 1994: 63–81.
7. Wahl LM, Wahl SM, Mergenhagen SE, Martin GR. Collagenase production by endotoxin activated macrophages. *Proc Natl Acad Sci USA* 1974; **71**: 3598–3601.
8. Feuerstein G, Hallenbeck JM, Vanatt AB, Rabinovici R, Perera PY, Vogel SN. Effect of gram-negative endotoxin on levels of serum corticosterone, TNFα, circulating blood cells, and the survival of rats. *Circ Shock* 1990; **30**: 265–278.
9. Klosterhafen B, Horstmenn-Jungemann K, Vogel P, Flohe S, Offner F, Kirckpatrick CJ, Heinrich PC. Time course of various inflammatory mediators during recurrent endotoxaemia. *Biochem Pharmacol* 1992; **43**: 2103–2109.
10. Mason MJ, Van Epps DE. In vivo neutrophil emigration in response to interleukin-1 and tumor necrosis factor. *J Leukoc Biol* 1989; **45**: 62–68.
11. Rampart MWDS, Fiers W, Herman AG. Inflammatory properties of recombinant tumor necrosis factor in rabbit skin in vivo. *J Exp Med* 1989; **169**: 2227–2232.
12. Harmsen AG, Havell EA. Roles of tumor necrosis factor and macrophages in lipopolysaccharide-induced accumulation of neutrophils in cutaneous air pouches. *Infect Immun* 1990; **58**: 297–302.
13. Klebanoff SJ, Vadas MA, Harlan JM, Sparks LH, Gamble JR, Agosti JM, Waltersdorph AM. Stimulation of neutrophils by tumor necrosis factor. *J Immunol* 1986; **136**: 4220.
14. Tsujimoto M, Yokota S, Vilceck J, Weismann G. Tumor necrosis factor provokes superoxide anion generation from neutrophils. *Biochem Biophys Res Commun* 1986; **137**: 1094.
15. Gamble JR, Harlan JM, Klebanoff SJ, Vadas MA. Stimulation of the adherence of neutrophils to umbilical vein endothelium by human recombinant tumor necrosis factor. *Proc Natl Acad Sci USA* 1985; **82**: 8667.
16. Ulich TR, Guo K, Del Castillo J. Endotoxin induced cytokine gene expression in vivo. *Am J Pathol* 1989; **134**: 11–14.
17. Beutler BA, Cerami AJ. Cachectin and tumor necrosis factor as two sides of the same biological coin. *Nature (Lond)* 1986; **320**: 584.
18. Ulich TR, Guo K, Irwin B, Remick DG, Davatelis GN. Endotoxin induced cytokine gene expression in vivo. II. Regulation of tumor necrosis factor and interleukin-1α/β expression and suppression. *Am J Pathol* 1990; **137**: 1173–1185.
19. de Castro CMMB, Bureau M-F, Vargaftig BB, Bachelet M. Interference of dexamethasone with leukocyte, blood volume and albumin movements in lungs from endotoxemics guinea-pigs. *Pulm Pharmacol* 1995; **8**: 289–297.
20. Ragsdale RL, Grasso RJ. An improved spectrofluorometric assay for quantifying yeast phagocytosis in cultures of murine peritoneal macrophages. *J Immunol Methods* 1989; **123**: 259–267.
21. Waage A. Production and clearance of tumor necrosis factor in rats exposed to endotoxin and dexamethasone. *Clin Immunol Immunopathol* 1987; **45**: 348–355.
22. Edwards JCW, Sedgwick AD, Willoughby DA. The formation of structure with the features of synovial lining by subcutaneous injection of air: an in vivo tissue culture system. *J Pathol* 1981; **134**: 147–156.
23. Weiss SJ. Tissue destruction by neutrophils. *N Engl J Med* 1989; **320**: 365–375.
24. Matsuda H, Kawakita K, Kiso Y, Nakano T, Kitamura Y. Substance P induces granulocyte infiltration through degranulation of mast cells. *J Immunol* 1989; **142**: 927–931.
25. Verbin RS, Goldblatt PJ, Farber E. The biochemical pathology of inhibition of protein synthesis in vivo: the effects of cycloheximide on hepatic parenchymal cell ultrastructure. *Lab Invest* 1969; **20**: 529–536.
26. Van Rooijen N, Van Nieuwmegen R. Elimination of phagocytic cells in the spleen after intravenous injection of liposome-encapsulated dichloromethylene diphosphonate. An enzyme-histochemical study. *Cell Tissue Res* 1984; **238**: 355–358.
27. Ledda-Columbano GM, Coni P, Faa G, Manenti G, Columbano A. Rapid induction of apoptosis in rat liver by cycloheximide. *Am J Pathol* 1992; **140**: 545–549.
28. Waring P. DNA fragmentation induced in macrophages by gliotoxin does not require protein synthesis and is preceded by raised inositol triphosphate levels. *J Biol Chem* 1990; **265**: 14476–14480.
29. Wyllie AH, Kerr JFR, Currie AR. Cell death: the significance of apoptosis. *Int Rev Cytol* 1980; **68**: 251–306.
30. Tepper CG, Studzinski GP. Teniposide induces nuclear but not mitochondrial DNA degradation. *Cancer Res* 1992; **52**: 3384–3390.
31. Gong J, Xun L, Darzynkiewicz Z. Different patterns of apoptosis of HL-60 cells induced by cycloheximide and camptothecin. *J Cell Physiol* 1993; **157**: 263–270.
32. Tannenbaum S, Oertel H, Henderson W, Kaliner M. The biologic activity of mast cell granules. I. Elicitation of inflammatory responses in rat skin. *J Immunol* 1980; **125**: 325–335.
33. Raud J, Dahlen SE, Smedegard G, Hedqvist P. An intra-vital microscopic model for mast cell-dependent inflammation in the hamster cheek pouch. *Acta Physiol Scand* 1989; **135**: 95–105.
34. Faccioli LH, Souza GEP, Cunha FQ, Poole S, Ferreira SH. Recombinant interleukin-1 and tumor necrosis factor induce neutrophil migration 'in vivo' by indirect mechanisms. *Agents Actions* 1990; **30**: 344–349.
35. Tachibana K, Chen G-J, Huang DS, Scuderi P, Watson RR. Production of tumor necrosis factor α by resident and activated murine macrophages. *J Leukoc Biol* 1992; **51**: 251–255.
36. Van Otteren GM, Standiford TJ, Kunkel SL, Danforth JM, Burdick MD, Abruzzo LV, Strieter RM. Expression and regulation of macrophage inflammatory-1α by murine alveolar and peritoneal macrophages. *Am J Respir Cell Mol Biol* 1994; **10**: 8–15.
37. Van Rooijen N, Kors N, Kraal G. Macrophage subset repopulation in the spleen: differential kinetics after liposome-mediated elimination. *J Leukoc Biol* 1989; **45**: 97–104.
38. Van Rooijen N, Kors N, Ende M, Dijkstra CD. Depletion and repopulation of macrophages in spleen and liver of the rat after intravenous treatment with liposome-encapsulated dichloromethylene diphosphonate. *Cell Tissue Res* 1990: **260**: 215–222.
39. Bogers WMJM, Stad R-K, Janssen DJ, Van Rooijen N, Van Es LA, Daha MR. Kuppfer cell depletion in vivo results in preferential elimination of IgG aggregates and immune complexes via specific Fc receptors on rat liver endothelial cells. *Clin Exp Immunol* 1995; **86**: 328.
40. Salkowski CA, Neta R, Wynn T, Strassman G, Van Rooijen N, Vogel SN. Effect of liposome-mediated macrophage depletion on LPS-induced cytokine gene expression and radioprotection. *J Immunol* 1995; **155**: 3168–3179.
41. Gordon JR, Galli SJ. Mast cells as a source of both preformed and immunologically inducible TNFα/cachectin. *Nature (Lond)* 1990; **346**: 274–276.
42. Matthews N. Tumor-necrosis factor from the rabbit. II. Production by monocytes. *Br J Cancer* 1978; **38**: 310.
43. Beutler BA, Mulsark IW, Cerami A. Cachectin/tumor necrosis factor: production, distribution and metabolic fate in vivo. *J Immunol* 1985; **135**: 3972–3977.
44. Chrisman JW, Petras SF, Vacek PM, Davis GS. Rat alveolar macrophage production of chemoattractants for neutrophils: response to *Escherichia coli* endotoxin. *Infect Immun* 1989; **57**: 810–816.
45. Windsor ACJ, Walsh CJ, Mullen PG, Cook DJ, Fisher BJ, Blocher CR, Leeper-Woodford SK, Sugerman HJ, Fowler III AA. Tumor necrosis factor-α blockade prevents neutrophil CD18 receptor and attenuates acute lung injury in porcine sepsis without inhibition of neutrophil oxygen radical generation. *J Clin Invest* 1993; **91**: 1459–1468.
46. Smart SJ, Casale TB. Pulmonary epithelial cells facilitate TNFα-induced neutrophil chemotaxis. *J Immunol* 1994; **152**: 4087–4094.
47. Furie MB, McHugh DD. Migration of neutrophils across endothelial monolayers is stimulated by treatment of the monolayers with IL-1 or tumor necrosis factor-α. *J Immunol* 1989; **143**: 3309.

**Received 5 June 1997;**
**accepted in revised form 1 July 1997**

## References

[PDF_01049] Beutler B. A., Milsark I. W., Cerami A. (1985). Cachectin/tumor necrosis factor: production, distribution, and metabolic fate in vivo.. J Immunol.

[PDF_00968] Beutler B., Cerami A. (1986). Cachectin and tumour necrosis factor as two sides of the same biological coin.. Nature.

[PDF_01036] Bogers W. M., Stad R. K., Janssen D. J., van Rooijen N., van Es L. A., Daha M. R. (1991). Kupffer cell depletion in vivo results in preferential elimination of IgG aggregates and immune complexes via specific Fc receptors on rat liver endothelial cells.. Clin Exp Immunol.

[PDF_01052] Christman J. W., Petras S. F., Vacek P. M., Davis G. S. (1989). Rat alveolar macrophage production of chemoattractants for neutrophils: response to Escherichia coli endotoxin.. Infect Immun.

[PDF_00919] Cybulsky M. I., McComb D. J., Movat H. Z. (1988). Neutrophil leukocyte emigration induced by endotoxin. Mediator roles of interleukin 1 and tumor necrosis factor alpha 1.. J Immunol.

[PDF_00984] Edwards J. C., Sedgwick A. D., Willoughby D. A. (1981). The formation of a structure with the features of synovial lining by subcutaneous injection of air: an in vivo tissue culture system.. J Pathol.

[PDF_01019] Faccioli L. H., Souza G. E., Cunha F. Q., Poole S., Ferreira S. H. (1990). Recombinant interleukin-1 and tumor necrosis factor induce neutrophil migration "in vivo" by indirect mechanisms.. Agents Actions.

[PDF_00939] Feuerstein G., Hallenbeck J. M., Vanatta B., Rabinovici R., Perera P. Y., Vogel S. N. (1990). Effect of gram-negative endotoxin on levels of serum corticosterone, TNF alpha, circulating blood cells, and the survival of rats.. Circ Shock.

[PDF_01062] Furie M. B., McHugh D. D. (1989). Migration of neutrophils across endothelial monolayers is stimulated by treatment of the monolayers with interleukin-1 or tumor necrosis factor-alpha.. J Immunol.

[PDF_00962] Gamble J. R., Harlan J. M., Klebanoff S. J., Vadas M. A. (1985). Stimulation of the adherence of neutrophils to umbilical vein endothelium by human recombinant tumor necrosis factor.. Proc Natl Acad Sci U S A.

[PDF_01010] Gong J., Li X., Darzynkiewicz Z. (1993). Different patterns of apoptosis of HL-60 cells induced by cycloheximide and camptothecin.. J Cell Physiol.

[PDF_01044] Gordon J. R., Galli S. J. (1990). Mast cells as a source of both preformed and immunologically inducible TNF-alpha/cachectin.. Nature.

[PDF_00953] Harmsen A. G., Havell E. A. (1990). Roles of tumor necrosis factor and macrophages in lipopolysaccharide-induced accumulation of neutrophils in cutaneous air pouches.. Infect Immun.

[PDF_00924] Hugli T. E. (1989). Chemotaxis.. Curr Opin Immunol.

[PDF_00916] Issekutz A. C., Bhimji S. (1982). Role for endotoxin in the leukocyte infiltration accompanying Escherichia coli inflammation.. Infect Immun.

[PDF_00956] Klebanoff S. J., Vadas M. A., Harlan J. M., Sparks L. H., Gamble J. R., Agosti J. M., Waltersdorph A. M. (1986). Stimulation of neutrophils by tumor necrosis factor.. J Immunol.

[PDF_00943] Klosterhalfen B., Hörstmann-Jungemann K., Vogel P., Flohé S., Offner F., Kirkpatrick C. J., Heinrich P. C. (1992). Time course of various inflammatory mediators during recurrent endotoxemia.. Biochem Pharmacol.

[PDF_01000] Ledda-Columbano G. M., Coni P., Faa G., Manenti G., Columbano A. (1992). Rapid induction of apoptosis in rat liver by cycloheximide.. Am J Pathol.

[PDF_00947] Mason M. J., Van Epps D. E. (1989). In vivo neutrophil emigration in response to interleukin-1 and tumor necrosis factor-alpha.. J Leukoc Biol.

[PDF_00989] Matsuda H., Kawakita K., Kiso Y., Nakano T., Kitamura Y. (1989). Substance P induces granulocyte infiltration through degranulation of mast cells.. J Immunol.

[PDF_01047] Matthews N. (1978). Tumour-necrosis factor from the rabbit. II. Production by monocytes.. Br J Cancer.

[PDF_00978] Ragsdale R. L., Grasso R. J. (1989). An improved spectrofluorometric assay for quantitating yeast phagocytosis in cultures of murine peritoneal macrophages.. J Immunol Methods.

[PDF_00950] Rampart M., De Smet W., Fiers W., Herman A. G. (1989). Inflammatory properties of recombinant tumor necrosis factor in rabbit skin in vivo.. J Exp Med.

[PDF_01040] Salkowski C. A., Neta R., Wynn T. A., Strassmann G., van Rooijen N., Vogel S. N. (1995). Effect of liposome-mediated macrophage depletion on LPS-induced cytokine gene expression and radioprotection.. J Immunol.

[PDF_01060] Smart S. J., Casale T. B. (1994). Pulmonary epithelial cells facilitate TNF-alpha-induced neutrophil chemotaxis. A role for cytokine networking.. J Immunol.

[PDF_01022] Tachibana K., Chen G. J., Huang D. S., Scuderi P., Watson R. R. (1992). Production of tumor necrosis factor alpha by resident and activated murine macrophages.. J Leukoc Biol.

[PDF_01013] Tannenbaum S., Oertel H., Henderson W., Kaliner M. (1980). The biologic activity of mast cell granules. I. Elicitation of inflammatory responses in rat skin.. J Immunol.

[PDF_01008] Tepper C. G., Studzinski G. P. (1992). Teniposide induces nuclear but not mitochondrial DNA degradation.. Cancer Res.

[PDF_00959] Tsujimoto M., Yokota S., Vilcek J., Weissmann G. (1986). Tumor necrosis factor provokes superoxide anion generation from neutrophils.. Biochem Biophys Res Commun.

[PDF_00970] Ulich T. R., Guo K. Z., Irwin B., Remick D. G., Davatelis G. N. (1990). Endotoxin-induced cytokine gene expression in vivo. II. Regulation of tumor necrosis factor and interleukin-1 alpha/beta expression and suppression.. Am J Pathol.

[PDF_00966] Ulich T. R., Guo K., del Castillo J. (1989). Endotoxin-induced cytokine gene expression in vivo. I. Expression of tumor necrosis factor mRNA in visceral organs under physiologic conditions and during endotoxemia.. Am J Pathol.

[PDF_01032] Van Rooijen N., Kors N., vd Ende M., Dijkstra C. D. (1990). Depletion and repopulation of macrophages in spleen and liver of rat after intravenous treatment with liposome-encapsulated dichloromethylene diphosphonate.. Cell Tissue Res.

[PDF_00992] Verbin R. S., Goldblatt P. J., Farber E. (1969). The biochemical pathology of inhibition of protein synthesis in vivo. The effects of cycloheximide on hepatic parenchymal cell ultrastructure.. Lab Invest.

[PDF_00981] Waage A. (1987). Production and clearance of tumor necrosis factor in rats exposed to endotoxin and dexamethasone.. Clin Immunol Immunopathol.

[PDF_00936] Wahl L. M., Wahl S. M., Mergenhagen S. E., Martin G. R. (1974). Collagenase production by endotoxin-activated macrophages.. Proc Natl Acad Sci U S A.

[PDF_01003] Waring P. (1990). DNA fragmentation induced in macrophages by gliotoxin does not require protein synthesis and is preceded by raised inositol triphosphate levels.. J Biol Chem.

[PDF_00929] Weingarten R., Sklar L. A., Mathison J. C., Omidi S., Ainsworth T., Simon S., Ulevitch R. J., Tobias P. S. (1993). Interactions of lipopolysaccharide with neutrophils in blood via CD14.. J Leukoc Biol.

[PDF_00987] Weiss S. J. (1989). Tissue destruction by neutrophils.. N Engl J Med.

[PDF_01055] Windsor A. C., Walsh C. J., Mullen P. G., Cook D. J., Fisher B. J., Blocher C. R., Leeper-Woodford S. K., Sugerman H. J., Fowler A. A. (1993). Tumor necrosis factor-alpha blockade prevents neutrophil CD18 receptor upregulation and attenuates acute lung injury in porcine sepsis without inhibition of neutrophil oxygen radical generation.. J Clin Invest.

[PDF_00925] Wright S. D., Ramos R. A., Hermanowski-Vosatka A., Rockwell P., Detmers P. A. (1991). Activation of the adhesive capacity of CR3 on neutrophils by endotoxin: dependence on lipopolysaccharide binding protein and CD14.. J Exp Med.

[PDF_01006] Wyllie A. H., Kerr J. F., Currie A. R. (1980). Cell death: the significance of apoptosis.. Int Rev Cytol.

[PDF_00974] de Castro C. M., Bureau M. F., Vargaftig B. B., Bachelet M. (1995). Interference of dexamethasone with leukocyte blood volume and albumin movements in lungs from endotoxemic guinea-pigs.. Pulm Pharmacol.

[PDF_01029] van Rooijen N., Kors N., Kraal G. (1989). Macrophage subset repopulation in the spleen: differential kinetics after liposome-mediated elimination.. J Leukoc Biol.

[PDF_00996] van Rooijen N., van Nieuwmegen R. (1984). Elimination of phagocytic cells in the spleen after intravenous injection of liposome-encapsulated dichloromethylene diphosphonate. An enzyme-histochemical study.. Cell Tissue Res.

